# The effect of sevoflurane anesthesia for dental procedure on neurocognition in children: a prospective, equivalence, controlled trial

**DOI:** 10.1186/s12887-021-02649-5

**Published:** 2021-04-16

**Authors:** Pinping Zhou, Chao Zhang, Guijin Huang, Yuan Hu, Wenzhu Ma, Cong Yu

**Affiliations:** 1 College of Stomatology, Chongqing Medical University, Chongqing, China; 2 Chongqing Key Laboratory for Oral Diseases and Biomedical Sciences, Chongqing, China; 3 Chongqing Municipal Key Laboratory of Oral Biomedical Engineering of Higher Education, Chongqing, China; 4 Department of Anesthesiology, Stomatological Hospital of Chongqing Medical University, Chongqing, China; 5 Department of Pediatric Dentistry, Stomatological Hospital of Chongqing Medical University, Chongqing, China; 6 Stomatological Hospital of Chongqing Medical University, 426 Songshi North Road, Yubei District, Chongqing, 401147 China

**Keywords:** Sevoflurane, General anesthesia, Dental procedure, Children, Neurocognition

## Abstract

**Background:**

Dental procedures under general anesthesia (DGA) was found to improve the oral health-related quality of children’s life. However, some parents and pediatricians expressed concern about the neurotoxicity of general anesthesia. The purpose of this trial was to whether DGA in children has an adverse effect on neurocognition.

**Methods:**

In this prospective, assessor-masked, controlled, equivalence trial, we recruited 340 children younger than 7 years who were undergoing caries treatment between Feb 1, 2019, and Aug 31, 2019, without factors affecting neurodevelopment. They received either sevoflurane-based general anesthesia or awake-local anesthesia. The Wechsler Preschool and Primary Scale of Intelligence-Fourth Edition was used to evaluate the neurocognitive function of children at 6 months after surgery, and the Full-Scale IQ (FSIQ) was selected as the primary outcome. The predefined clinical equivalence margin was 5 (1/3 SD of FSIQ score). If the 95% CI of the difference between the average FSIQ score of the two groups is within − 5 to + 5, then the two groups are equivalent.

**Results:**

The outcome data were obtained from 129 children in the general anesthesia group and 144 in the local anesthesia group. The median length of general anesthesia was 130 min (IQR 110–160). The mean FSIQ score in the general anesthesia group was 103·12 (SD 8.94), and the mean of the local anesthesia group was 103·58 (SD 8.40). There was equivalence in means of FSIQ score between the two groups (local minus general anesthesia 0.46, 95% CI − 2.35 to 1.61). There was no significant difference in FSIQ scores between different age groups and different anesthesia durations. Only the mother’s education could affect the primary outcome.

**Conclusions:**

In this trial, prolonged DGA with a sevoflurane-only anesthetic in preschool children, does not adversely affect neurocognitive function at 6 months after surgery compared with awake-local anesthesia.

**Trial registration:**

Chinese Clinical Trial Registry, ChiCTR1800015216. Registered Mar 15 2018.

## Background

Recent studies have found that the prevalence of caries in children aged 3 to 5 years was 50.8 to 71.9% in China [1]. A growing number of children have caries of multiple teeth, and the treatment time is longer, usually more than 1 h. For children with severe dental anxiety (DA), dental procedures under general anesthesia (DGA) is common practice for anesthesia for caries treatment. DGA has been demonstrated to have a higher success rate and safety [2, 3]. In our previous research, we found that DGA with sevoflurane improved the oral health-related quality of children’s life, and most families expressed high satisfaction [4].

However, many parents and pediatricians were still concerned about the neurotoxicity of general anesthesia found in animal models [5–9] and hesitated whether to delay the procedure. Practically, several noted clinical studies [10–15] did not find that general anesthesia harmed the development of children. For example, in the GAS study, it was found that infants who underwent inguinal hernia repair under general anesthesia with sevoflurane did not have the adverse neurodevelopmental outcome. The PANDA study took children who had undergone inguinal hernia repair under general anesthesia before the age of 3 years as the research object and matched their healthy siblings. It also found that anesthesia had no negative effects on the children. Currently, there was no relevant research in the field of dental procedure, and these clinical studies used multiple anesthetics or had a shorter duration of surgery.

Therefore, the purpose of our trial was to investigate the effect of prolonged exposure to sevoflurane for DGA on the neurocognitive function in healthy preschool children.

## Methods

### Study design

We did a prospective, controlled, assessor-blind, equivalence trial comparing neurocognitive outcomes at 6 months after surgery, in preschool children undergoing caries treatment under sevoflurane-based general anesthesia or awake-local anesthesia. The trial was done at the Stomatological Hospital of Chongqing Medical University, and the written consent was obtained from parents or guardians. The study had been treated according to the declaration of Helsinki. It was registered with the Chinese Clinical Trial Registry (number ChiCTR1800015216), and the ethics committee approval of the Stomatological Hospital of Chongqing Medical University was obtained (number CQHS-IRB-2018-01).

Children’s neurocognitive function 6 months after surgery was assessed by the Wechsler Preschool and Primary Scale of Intelligence, fourth edition (WPPSI-IV) Chinese version (CN). The WPPSI-IV (CN) is a test scale used to assess general intellectual function and neurocognitive development. It is mainly used in clinical and research fields, and it has a high-quality norm in China and high clinical validity. There are extensive academic literature and application experience about the WPPSI-IV (CN). The WPPSI-IV (CN) includes 13 subtests, and the composite score includes the full-scale intelligence quotient (FSIQ), main index, and auxiliary index. The primary outcome for the analysis on a per-protocol basis was prespecified to be the FSIQ. The secondary outcomes for this analysis were selected main indexes, including Verbal Comprehension Index (VCI), Visual-Spatial Index (VSI), Fluid Reasoning Index (FRI), Working Memory Index (WMI), and Processing Speed Index (PSI), which are mainly used to evaluate the ability of knowledge expression and spoken language, visual-spatial information processing and vision-action coordination, fluid intelligence and inductive reasoning, visual working memory and attention, and cognitive flexibility and visual scan.

The sample size of this trial was calculated based on the FSIQ score. The mean of the FSIQ score in the reference population is 100, and the standard deviation is 15. Assuming a sample ratio of 1: 1, a significance level of 0.05, and a test power of 80%, the trial would need 308 patients. Enrolling roughly 340 patients would allow for 10% of dropping out. So, we planned to recruit 170 children receiving general anesthesia and 170 children receiving local anesthesia for dental procedures. The first assessment was performed six months after surgery. The anesthetists, pediatricians, and parents were aware of group allocation, but the researchers who administered the neurofunctional assessments were not.

### Participants

Inclusion criteria included healthy children less than 7 years old scheduled for caries treatment, and children classified as ASA I. According to the Frankl’s Behavior Rating Scale (FBRS), children with rating 1 (definitely negative behavior) received sevoflurane-based general anesthesia, and children with rating 2 to 4 (negative, positive, and definitely positive behavior) received awake-local anesthesia.

Exclusion criteria were any contraindication for anesthesia, previous exposure to general anesthesia, moderate to severe preterm infants (gestational age no more than 33 weeks), very low birth weight infant (birth weight less than 1500 g), epileptic, any known neurological injury or developmental issues, other known diseases that might affect neurodevelopment, deaf-mute or blind, and any reason that might make follow-up difficult. Eligible children were recruited in the Department of Pediatric Dentistry or Anesthesiology of the Stomatological Hospital of Chongqing Medical University. During the research, children were excluded if they suffered brain injuries, underwent any other surgery, or failed to complete all procedures as protocol.

### Anesthesia procedures

The awake-local anesthesia group received the local infiltration anesthesia after the behavior induction. Children under 4 years old were given 2% lidocaine hydrochloride injection (5 ml: 0.1 g, Southwest Pharmaceutical Co.; SFDA no.H50020038), with the total dose no more than 4 mg/Kg. Children aged 4 years and older were given 4% articaine hydrochloride and epinephrine tartrate injection (1.7 ml: 68 mg, Produits Dentaires Pierre Rolland; SFDA no.H20140732), with the maximum dosage not exceeding 5 mg/Kg.

The general anesthesia group only received sevoflurane (120 ml, Shanghai Hengrui Pharmaceutical Co.; SFDA no.H20070172) for induction and maintenance. After induction with 5 L / min oxygen and 8% sevoflurane through the mask, the anesthetists chose and inserted the modified first-generation single-lumen laryngeal mask airway of the appropriate model. Anesthetists adjusted sevoflurane concentration during maintenance according to vital signs and Bispectral Index (the majority between 40 and 60 for the suitable depth of anesthesia), mostly 2.5 to 3.5% with a 2 L / min mixture of air and oxygen. In the whole process, the patients retained spontaneous breathing, and PetCO_2_ was usually maintained within 35 to 45 mmHg. Supplemental opioids, benzodiazepines, and other general anesthetics were not allowed, but local anesthetics were permitted to provide analgesia with the same dose as the local anesthesia group. There was postanesthesia care for 2 h after surgery. They were allowed to leave the hospital after the modified Aldrete score reached 10 points.

### Data collection and measure

Before the operation, demographic data, pregnancy and birth details, and family structure were collected. During surgery, anesthetists recorded vital signs and perioperative adverse events. The patients were followed up by telephone on the day of surgery and the first day after surgery.

At 6 months after surgery, the WPPSI-IV (CN) was used to assess the neurocognitive function for two groups of children. It was to be done within 1 month after half a year after surgery. All children individually completed one-to-one with the qualified assessor. It took approximately 1.5 h for each child to complete the assessment. Parents were asked if children had been found physical abnormalities since caries treatment, and brief physical and neurological examinations were done for patients.

### Statistical analysis

The hypothesis was that the FSIQ score is equivalent in children who have received general anesthesia or local anesthesia for caries treatment 6 months after surgery. The equivalence margin was defined as five (1/3 SD), and a two one-sided test was performed with the 0.025 level of significance. Equivalence was predefined as the 95% CI of difference between the mean of the FSIQ score within − 5 to + 5. The secondary outcomes, VCI, VSI, FRI, WMI, and PSI, also have a mean of 100 and a standard deviation of 15. It is reasonable to assume that their equivalence margins are five. All CIs are two-sided.

The statistical analysis plan prespecified the age at evaluation (< 4 years old or ≥ 4 years old) as a subgroup analysis. Several predictor variables of primary outcome used in the regression model included anesthesia group, sex, gestational age at birth, birthweight, past medical histories of children, mother’s abnormalities during pregnancy, mother’s education, maternal age at delivery, perioperative adverse events, age at evaluation, and postoperative developmental disorders. In the general anesthesia group, the duration of sevoflurane exposure was used as a predictor variable and grouped (less than 120 min, 120 to 180 min, and more than 180 min) to observe the effect of different anesthesia duration on the primary outcome. All analyses were done in SPSS (version 25).

## Results

### Trial process

Between Feb 1, 2019, and Aug 31, 2019, 1681 children were screened for eligibility, and 340 patients were recruited. After three withdrawal of consent by parents (pre-surgery), the intent-to-treat analysis included 168 children in the general anesthesia group and 169 children in the local anesthesia group. Thirty-one participants with surgery being canceled or protocol violations were excluded, and 150 patients in the general anesthesia completed the surgery and 156 in the local anesthesia group.

**Fig. 1:**
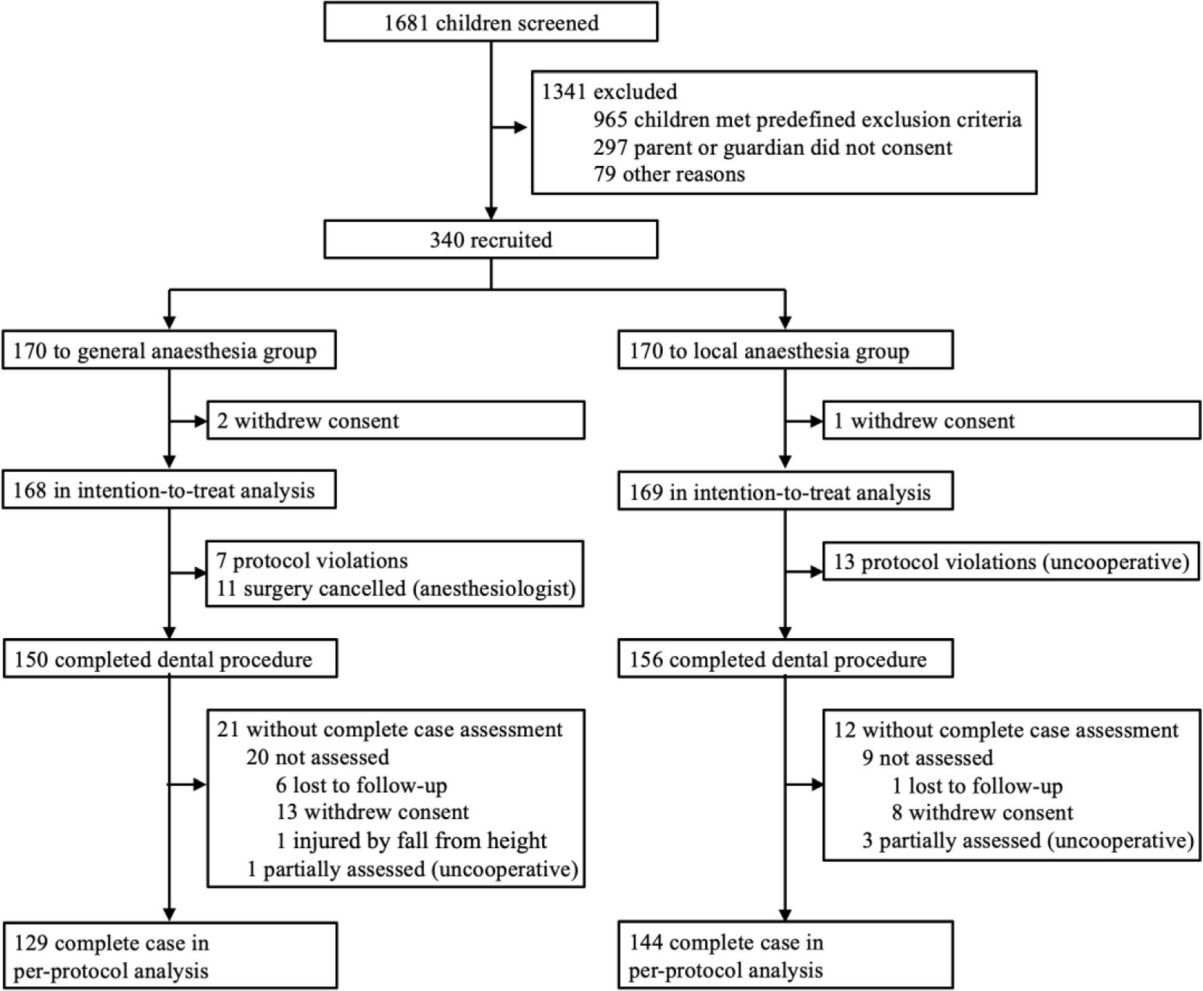
Trial profile

Follow-up was from Aug 1, 2019, to Mar 22, 2020. Twenty-eight families were lost to follow-up or withdrew consent, and one child was injured by falling from a height. In the per-protocol analysis, the WPPSI-IV (CN) was complete for 129 in the general anesthesia group and 144 in the local anesthesia group (Fig. 1).

### Demographic data at baseline and non-scale measurement after surgery

Demographic data at baseline and anesthesia details are shown in Table 1. There were 63 boys (49%) in the general anesthesia group and 77 boys (53%) in the local anesthesia group. The age at surgery was from 2.5 to 6.5 years old. Among all participants, 9 mothers had mild abnormalities during pregnancy, and 12 children had a past medical history. The median duration of sevoflurane use was 130 min (110 to 160). The only adverse event during the perioperative period in the general anesthesia group was related to respiratory complications (two cases of slight laryngospasm), and one case of the local anesthetic adverse reaction occurred in the local anesthesia group.

**Table 1:** Demographic data at baseline and anesthesia details

	GA group (***n*** = 129)	LA group (***n*** = 144)	***P*** vaule^**a**^
**Baseline demographics**			
Sex, M/F	63/66	77/67	0.444
Age at surgery			
< 4 years old	36	22	0.011
≥ 4 years old	93	122	
Weight, kg; median (IQR)	17.50 (15.80–18.70)	18.35 (16.50–20.20)	< 0.001
Height, cm; median (IQR)	106.00 (99.50–110.00)	106.00 (101.00–115.00)	0.003
Past medical history			
Non-recurrent febrile convulsion	2 (2%)	3 (2%)	1.000
Thalassemia without a history of blood transfusion	3 (2%)	0	0.104
Favism	0	1 (<1%)	1.000
Patent ductus arteriosus without treatment	2 (2%)	1 (<1%)	0.604
**Pregnancy and birth details**			
Preterm (born < 37 weeks’ gestation)	8 (6%)	5 (3%)	0.290
Birthweight, kg	3.30 (0.53)	3.29 (0.46)	0.909
One of a multiple pregnancy	2 (2%)	1 (<1%)	0.604
Abnormal pregnancy examination of mother	4 (3%)	5 (3%)	1.000
Delivery mode			
Cephalic vaginal	62 (48%)	73 (51%)	0.664
Cesarean section with intravertebral anesthesia	67 (52%)	71 (49%)	
**Family demographics**			
Maternal age at birth more than 30 years	33 (26%)	45 (31%)	0.302
Maternal education			
Junior high school	7 (5%)	5 (3%)	0.486
Senior high school	31 (24%)	27 (19%)	
Bachelor degree	82 (64%)	108 (75%)	
Master degree or higher	9 (7%)	4 (3%)	
Number of children in the family			
1	83 (64%)	92 (64%)	0.909
2	45 (35%)	50 (35%)	
3	1 (<1%)	2 (1%)	
Birth order			
1	108 (84%)	114 (79%)	0.324
2	21 (16%)	29 (20%)	
3	0	1 (<1%)	
**Anaesthesia details**			
Duration of surgery, min; median (IQR)	115 (95–140)	NA	
Duration of use of sevoflurane, min; median (IQR)	130 (110–160)	NA	
Adverse events related to the cardiovascular system	0	1 (<1%)^b^	1.000
Adverse events related to the respiratory system	2 (2%)^c^	0	0.222
Apparent hypoxia^d^	0	0	

Data are presented as n, means (standard deviations), or median (IQR)

*GA* General anesthesia, *LA* Local anesthesia

^a^Pearson’s chi-squared test, Fisher’s exact test, Rank sum test, or Two independent samples t-test

^b^One patient had an adverse reaction of local anesthetic with increased heart rate (recovered after treatment)

^c^Two children had mild laryngospasm without evident hypoxia during anesthesia recovery

^d^Apparent hypoxia defined as oxygen saturation < 90%

Non-scale measurements at 6 months after surgery are shown in Table 2. There were 16 children less than 4 years old in the general anesthesia group and 9 children in the local anesthesia group. After surgery, no child had a diagnosis of febrile convulsion, epileptic seizure, or cerebral palsy, nor did they fail to complete WPPSI-IV (CN) due to developmental issues or behavioral disorders. The neurological examinations of all children were normal.

**Table 2:** Non-scale measurement at 6 months after surgery

	GA group (***n*** = 129)	LA group (***n*** = 144)	***P*** vaule^**a**^
**Assessment details**			
Age at follow-up assessment			
< 4 years old	16	9	0.078
≥ 4 years old	113	135	
Weight, kg; median (IQR)	19.60 (17.90–20.85)	20.20 (18.60–22.10)	0.003
Height, cm; median (IQR)	110.00 (103.00–114.00)	110.00 (106.00–119.00)	0.005
Abnormal neurological examinations	0	0	
**Events after caries treatment**			
Febrile convulsion or epileptic seizure	0	0	
Cerebral palsy	0	0	
Developmental delay	0	0	
Hearing or vision impairment	0	0	
Language, behavior or psychomotor disorder	0	0	
Intervention for neurodevelopmental problem	0	0	

Data are presented as n or median (IQR)

*GA* General anesthesia, *LA* Local anesthesia

^a^Pearson’s chi-squared test or Two independent samples t-test

### Overall results of scale measurement

The scores of FSIQ and main indexes (VCI, VSI, FRI, WMI, and PSI) in the WPPSI-IV (CN) of the two groups were shown in Table 3. For the FSIQ score, the mean was 103·12 (SD 8.94) in the general anesthesia group and 103·58 (8.40) in the local anesthesia group. We noted evidence for equivalence between groups in means (mean difference for local anesthesia minus general anesthesia 0.46, 95% CI − 2.35 to 1.61). There was also evidence for equivalence in the scores of VCI, VSI, FRI, WMI, and PSI between groups. The upper and lower limits of the 95% CIs were well inside the prespecified equivalence margin of five in all analyses.

**Table 3:** Comparison of WPPSI-IV(CN) scores between groups

Composite score	GA group (***n*** = 129)	LA group (***n*** = 144)	Difference in LA-GA	95% CI for difference in LA-GA
FSIQ	103.12 (8.94)	103.58 (8.40)	0.46	−2.53 to 1.61
VCI	105.57 (10.72)	104.81 (13.61)	−0.76	−2.18 to 3.70
VSI	102.12 (13.30)	104.09 (10.73)	1.97	−4.84 to 0.90
FRI	105.44 (9.89)^a^	106.07 (12.33)^b^	0.63	−3.46 to 2.20
WMI	101.10 (11.28)	101.64 (11.58)	0.54	− 3.27 to 2.19
PSI	106.71 (9.96)^a^	105.46 (11.02)^b^	−1.25	−1.40 to 3.90

Data are means (SD)

*GA* General anesthesia, *LA* Local anesthesia, *WPPSI-IV(CN)* Wechsler Preschool and Primary Scale of Intelligence-Fourth Edition (Chinese version), *FSIQ* Full Scale IQ, *VCI* Verbal Comprehension Index, *VSI* Visual Spatial Index, *FRI* Fluid Reasoning Index, *WMI* Working Memory Index, *PSI* Processing Speed Index.

^a^There is no FRI and PSI in the age group of less than 4 years (*n* = 113)

^b^There is no FRI and PSI in the age group of less than 4 years (*n* = 135)

### Subgroup analysis and predictor variables analysis of scale measurement

In the subgroup analysis, the FSIQ score between groups was equivalent in the age group less than 4 years old. It was the same in the age group older than 4 years. There was no significant difference between the two age groups in the general anesthesia group and the local anesthesia group (*p* > 0.05). For the secondary outcomes, in the age subgroup of younger than 4 years, the VSI and WMI scores were not found to be equivalent between groups; in the age subgroup of older than 4 years, the VSI score was not equivalent (Table 4).

**Table 4:** Comparison of WPPSI(CN) scores in age subgroups

Composite score	< 4 years old in GA group (***n*** = 16)	< 4 years old in LA group (***n*** = 9)	Difference (< 4 years old) in LA-GA (95% CI)	≥ 4 years old in GA group (***n*** = 113)	≥ 4 years old in LA group (***n*** = 135)	Difference (≥ 4 years old) in LA-GA (95% CI)
FSIQ	101.56 (4.72)	102.33 (5.20)	0.77 (−4.99 to 3.45)	103.58 (9.17)	103.67 (8.58)	0.09 (−2.31 to 2.13)
VCI	103.69 (5.49)	103.78 (5.14)	0.09 (−4.72 to 4.54)	105.26 (11.05)	104.70 (13.65)	−0.56 (−2.59 to 3.71)
VSI	103.56 (9.14)	98.11 (7.13)	−5.45 (−1.87 to 12.77)^b^	101.86 (13.64)	104.49 (10.83)	2.63 (−5.69 to 0.43)^b^
FRI	NA^a^	NA^a^		105.44 (9.89)	106.07 (12.33)	0.63 (−3.46 to 2.20)
WMI	104.75 (7.00)	105.78 (6.16)	1.03 (−6.82 to 4.76)^b^	100.60 (11.21)	101.67 (11.64)	1.07 (−3.94 to 1.80)
PSI	NA^a^	NA^a^		106.71 (9.96)	105.46 (11.02)	−1.25 (−1.40 to 3.90)

Data are means (SD)

*GA* General anesthesia, *LA* Local anesthesia, *WPPSI-IV(CN)* Wechsler Preschool and Primary Scale of Intelligence-Fourth Edition (Chinese version), *FSIQ* Full Scale IQ, *VCI* Verbal Comprehension Index, *VSI* Visual Spatial Index, *FRI* Fluid Reasoning Index, *WMI* Working Memory Index, *PSI* Processing Speed Index.

^a^There is no FRI and PSI in the age group of less than 4 years

^b^There is no equivalence

**Fig. 2:**
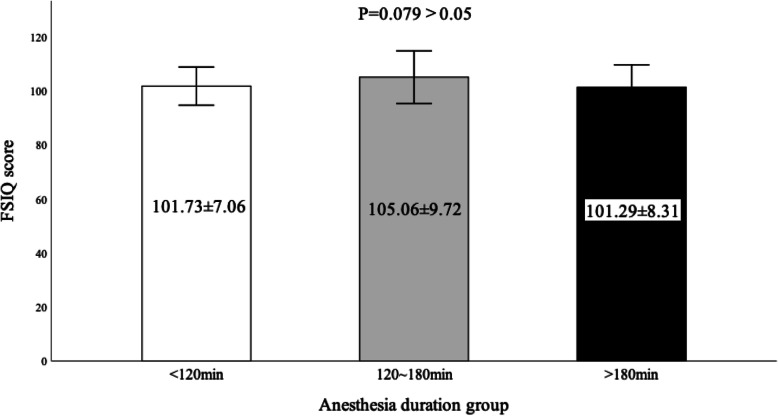
Comparison of the FSIQ scores between groups of anesthesia duration

In the unary linear regression analysis, it was found that the anesthesia group, sex, preterm, birthweight, past medical histories of children, mother’s pregnancy disease, maternal age at delivery, perioperative adverse events, and age at evaluation could not significantly affect the primary outcome (*p* > 0.05). Only the significance level of maternal education was less than 0.05 (b = 1.94, adjusted R-squared = 1.60%), which could affect the FSIQ score (Table 5). And from Table 1 we can see that there was no significant difference in the distribution of mother’s education between the two groups of children. However, after incorporating all the above predefined predictor variables into the multiple linear regression analysis, it was found that the overall significance level was above 0.05, and the significance of the mother’s education was also similar. It may be that although the mother’s education could affect the outcome in the unary linear regression, the influence was relatively small. When it was included in the multiple linear regression, the influence of maternal education was concealed due to the interaction between the predictor variables. In the general anesthesia group, the duration of sevoflurane exposure did not significantly affect the primary outcome (*p* = 0.860), and there was no significant difference in FSIQ score between groups (less than 120 min, 120 to 180 min, and more than 180 min) of different anesthesia durations (*p* = 0.079). (as shown in Fig. 2).

**Table 5:** Regression analysis of predictor variables

Predictor variables	***P*** vaule	b	R square
Anesthesia group	0.787		
Sex	0.263		
Preterm	0.694		
Birthweight	0.977		
Past medical histories of children	0.715		
Mother’s pregnancy disease	0.218		
Mother’s education	0.022^a^	1.941	1.60%
Maternal age at delivery	0.192		
Perioperative adverse events	0.916		
Age at evaluation	0.476		

^a^The significance level < 0.05

Besides, we also analyzed the delivery mode and family structure and found that the cesarean section and second child could not significantly affect the primary outcome (*p* > 0.05).

## Discussion

We noted evidence for equivalence between general anesthesia and local anesthesia in children for caries treatment in terms of the FSIQ in WPPSI-IV (CN) measured at 6 months after surgery. Equivalence was also shown in the VCI, VSI, FRI, WMI, and PSI, because the 95% CIs of the differences in means fell inside a third of an SD, well within our predefined margins of clinical equivalence. Local infiltration anesthesia in children is known to be safe and effective [16]. Therefore, it can be reasonably considered that children receiving prolonged DGA with sevoflurane alone had no adverse changes in neurocognitive development at 6 months after surgery.

The WPPSI-IV (CN) is a well-proven method for evaluating development, and the FSIQ score as the overall result is an appropriate evaluation indicator. Intelligence that covers multiple cognitive areas (memory, reasoning, spatial ability, executive ability, and processing speed) is deemed to be a relatively steady and constant trait of young children, and intellectual development in early childhood can predict and evaluate future intelligence [17]. Consequently, we use the WPPSI-IV (CN), a validated, internationally recognized, and standardized measurement tool to assess the neurocognitive development and general intellectual of children. It has neuropsychological evaluation characteristics and can provide composite scores representing the intellectual function of specified cognitive domains (verbal IQ, performance IQ, and processing velocity) and an overall score for FSIQ [18]. Two previous studies have used medical and school records to assess children exposed to anesthesia [19, 20]. However, individually measured cognitive abilities have proven to be more sensitive than the performance of group-tested academic skills [21].

The combination of disease and surgery made previous retrospective studies challenging to assess the independent effect of anesthesia exposure [20, 22]. In the PANDA study, more than one anesthetic was used [13]. The GAS trial was dominated by male infants (over 80%), and the duration of surgery was less than 1 h (median 54 min) [10, 11].

Therefore, this trial only included healthy children who had no potential factors or disease that influenced neurodevelopment. For the surgery, we selected caries treatment, which has little painful noxious stimulation and has almost no effect on the body’s internal environment. The general anesthesia group used a sevoflurane-only anesthetic. It was to reduce the interference of the confounding factors of the disease, surgery, and the drug interaction of multiple general anesthetics. Considering that behavioral differences after general anesthesia in animal models may be owing to sex differences [23], the ratio of male and female recruited was approximately 1:1. The GAS study has expounded the outcome of general anesthesia within 1 h. The aim of this trial was to assess the effects of prolonged (1 to 4 h) general anesthesia exposure on children (not just boys).

It was not a completely randomized trial. The choice of anesthesia method depended on the children’s behavioral rating during treatment based on the FBRS. Compared with general anesthesia, children who could undergo dental procedures under local anesthesia were usually older. Therefore, the two groups of children differ slightly in height and weight (Tables 1 and 2).

At present, there is no direct correlation between anesthesia and neurotoxicity of cognitive or behavioral sequelae in children [24]. Children undergoing surgery at a very young age are more likely to have underlying diseases, which may confuse the effects of general anesthesia exposure on neurodevelopmental outcomes [17]. Recent findings suggested that anesthetic neurotoxicity is not a major contributor to adverse neurodevelopmental outcomes for most healthy children who require surgery. Biological, environmental, and social factors are of far greater import [25]. In the subgroup analysis, we found that although the VSI and WMI scores in the secondary outcomes were not equivalent between groups. However, in the age subgroup of younger than 4 years, the average VSI score of the local anesthesia group minus the general anesthesia group was − 5.45, while the average WMI score of the local anesthesia group minus the general anesthesia group was 1.03 (Table 4). That is, it was not that the general anesthesia group’s score is always lower than that of the local anesthesia group. This difference might be caused by environmental or other factors, rather than general anesthesia. Therefore, these two evaluation indicators of the secondary outcomes couldn’t interfere with our conclusions.

We found that the mother’s education had an impact on the neurocognitive outcome in children. The higher the mother’s education, the better comprehensive neurocognitive ability on children, but the influence is not prominent. Previous studies have also found evidence that mother’s education could affect intellectual development and academic performance in children [14, 26]. Inconsistent with other research conclusions [14, 23], we did not find that premature, the duration of sevoflurane exposure, and the age of children had a significant effect on the neurocognitive outcome. It was similar in maternal abnormalities during pregnancy and past medical histories of children. It might be because after the screening, moderate to severe preterm infants were excluded, and these participants just had the mild disease in the past. They were relatively healthy, and we minimized the interference of confounding factors on the outcome.

Awake-local anesthesia inevitably has a failure rate. It was unavoidable that children may refuse surgery or change to general anesthesia due to DA. If all children were analyzed on an intention-to-treat basis, the results obtained would be more conservative and dilute the potential effect of general anesthesia, making the differences between groups more difficult to detect. To minimize this bias and ensure the reliability of the results, we analyzed mainly on a per-protocol basis.

Our trial has several limitations. The local anesthesia group could not compare the duration of surgery and anesthesia with the general anesthesia group. Although the two groups of children have similar severity of caries, almost all children with multiple caries could not complete all treatments at once with local anesthesia. It was usually required multiple treatments so that the duration could not be counted. And the general anesthesia group used a sevoflurane-only anesthetic without other general anesthetics. Thus, our trial cannot be generalized to practice in all pediatric surgery, and cannot elucidate possible outcomes if other agents are added. However, due to the local oral treatment, this type of treatment has little interference to the body’s internal environment, can quickly return to the preoperative state. The interference to the function of the central nervous system caused by the operation was eliminated, and the influence of anesthetic factors can be highlighted to the maximum extent. This is also the advantage of this study compared to other types of surgery.

Another limitation is that the children were recruited in urban areas of Chongqing, Southwest China, and no children from rural areas were recruited. The cultural education they have received is similar. The findings might be influenced by the differences from region and education. And there were fewer children under 4 years of age in the two groups of participants in this trial, and the conclusion in the study was mainly based on children over 4 years old. Because the trial recruited patients with long surgery duration to investigate the outcome of children after prolonged anesthesia. Younger children tended to have less serious caries and required shorter treatment time to meet the exclusion criteria, so there were fewer children under 4 years of age. It needs to be pointed out that, we did not evaluate the children before surgery. Currently, we only performed the first assessment of participants 6 months after surgery. Because the second assessment of WPPSI must be at least 1 year after the first assessment. Our research plan is to conduct the continuous evaluation of participants from short-term to long-term after surgery, in order to more accurately describe the changes in neurocognitive outcomes after DGA.

## Conclusions

This trial, being congruent with data from several previous clinical studies, found no evidence that prolonged exposure to DGA only with sevoflurane in relatively healthy preschool children causes adverse neurocognitive outcomes (including language, reasoning, memory, and vision-action) and neurological deficits at 6 months after surgery. Although this is not a final conclusion, these findings should reassure pediatricians and parents considering delaying surgery due to developmental disorders and medical risks. The longer-term neurocognitive outcomes after anesthesia exposure need to be assessed after 1, 2, and 5 years to draw conclusions.

## Data Availability

The datasets used and/or analysed during the current study are available from the corresponding author on reasonable request.

## Abbreviations

DGA: Dental procedures under general anesthesia
DA: Dental anxiety
FBRS: The Frankl’s Behavior Rating Scale
WPPSI (CN): The Wechsler Preschool and Primary Scale of Intelligence-Fourth Edition Chinese version
FSIQ: The Full-Scale IQ
VCI: Verbal Comprehension Index
VSI: Visual-Spatial Index
FRI: Fluid Reasoning Index
WMI: Working Memory Index
PSI: Processing Speed Index
